# Preclinical Female Model of Urogenital Dysfunction and Pathophysiological Changes After Pelvic Radiation Therapy

**DOI:** 10.7759/cureus.66374

**Published:** 2024-08-07

**Authors:** Bethlehem Peters, Shelby A Powers, Lindsey K Burleson, Michael R Odom, Elena S Pak, Alexander C Turner, Nethusan Sivanesan, Bridget F Koontz, Johanna L Hannan

**Affiliations:** 1 Department of Physiology, Brody School of Medicine, East Carolina University, Greenville, USA; 2 Department of Psychiatry and Behavioral Sciences, Duke University School of Medicine, Durham, USA; 3 Department of Obstetrics and Gynecology, Indiana University School of Medicine, Indianapolis, USA; 4 Department of Urology, Duke University School of Medicine, Durham, USA; 5 Department of Biomedical Sciences, Louisiana State University (LSU) School of Veterinary Medicine, Baton Rouge, USA; 6 Department of Medicine, University of Texas (UT) Southwestern Medical Center, Dallas, USA; 7 Department of Radiation Oncology, Brody School of Medicine, East Carolina University, Greenville, USA

**Keywords:** preclinical model, vaginal inflammation, bladder dysfunction, vaginal atrophy, female sexual dysfunction, pelvic radiation

## Abstract

Introduction

Radiation therapy (RT) is the gold standard for many pelvic cancers and improves overall patient survival. However, pelvic RT is associated with increased sexual dysfunction and urinary incontinence. Although the side effects of pelvic RT are well-documented, the pathological mechanisms leading to pelvic organ dysfunction are unknown, and a preclinical model has not been established. This study characterized the impact of pelvic RT at early and late timepoints on female rat bladder, vaginal, and urethral physiology and morphology.

Methods

Adult female Sprague-Dawley rats were divided into three groups (n = 8/group): (I) Sham, (II) four weeks RT (4wk RT), and (III) nine weeks RT (9wk RT). The RT groups received a single dose of 20 Gy external beam radiation, and experiments were conducted at 4wk and 9wk post-RT. Nerve-mediated vaginal blood flow was measured via laser Doppler. Tissue bath studies assessed vaginal contractility to electric field stimulation (EFS), adrenergic and cholinergic agonists, and relaxation to a nitric oxide donor. Bladder and urethral sphincters were evaluated for cholinergic, caffeine, and EFS-mediated contractility. Quantitative polymerase chain reaction (qPCR) measured gene expression of markers of oxidative stress. Vaginal, bladder, and urethral fibrosis were assessed with Masson’s trichrome staining.

Results

At 4wk post-RT, total vaginal blood flow decreased, and at 9wk post-RT, returned to baseline levels. At 9wk post-RT, vaginal neurogenic and adrenergic-mediated contractile responses increased significantly. Vaginal epithelial thickness decreased post-RT and correlated with an acute rise in vaginal inflammatory gene expression. At 4wk post-RT, bladder neurogenic contractions decreased and remained lowered. Internal urethral contractions increased at 4wk post-RT and returned to Sham levels after 9wk post-RT. Pelvic RT increased external urethral neurogenic contractions, which remained elevated.

Conclusion

This novel preclinical model provides valuable insights into the temporal pathophysiology of pelvic RT-induced sexual and urinary dysfunction. The establishment of this model is crucial for understanding the underlying mechanisms involved in RT-induced pelvic injury. A reliable, clinically relevant model will allow for the testing of therapeutic strategies to prevent adverse effects with RT in pelvic cancer survivors.

## Introduction

In 2020, more than two million women worldwide were diagnosed with pelvic cancers, including cervical, endometrial, colorectal, anal, bladder, and vulvar malignancies [1]. For pelvic cancers, radiotherapy (RT) is used to treat almost half of all patients [2]. Despite advancements in cancer therapies, a significant proportion of pelvic RT cancer survivors face long-term complications, specifically sexual dysfunction and urinary incontinence [3]. After RT, approximately 50-80% of endometrial, cervical, and anorectal cancer survivors experience vaginal stenosis, and up to 45% suffer from urinary toxicity [4-6]. These complications significantly impact the survivors’ quality of life.

Currently, there are no effective preventive strategies for pelvic RT-induced sexual dysfunction or urinary incontinence. While dilators are commonly used to prevent vaginal adhesions and stenosis, their effectiveness is limited due to poor patient adherence and insufficient evidence supporting standardized usage protocols [2,3,5]. Various treatments, including topical hormones, moisturizers, hyaluronic acid, and vitamins A and E, have been explored to manage RT-induced vaginal injuries, but their effectiveness remains uncertain [5]. Additionally, there is a lack of preventive measures for RT-induced urinary toxicity and subsequent incontinence. Given the scarcity and heterogeneity of existing evidence, further research is necessary to advance treatment options.

Vaginal stenosis and urinary incontinence are well-established complications of pelvic RT. The physical changes contributing to sexual dysfunction are well documented and consist of vaginal narrowing, loss of elasticity, adhesions, decreased vaginal secretions, and fistula development [2,7,8]. However, knowledge regarding female urethral and bladder toxicity following pelvic RT is limited. Pelvic cancer survivors report symptoms of increased frequency, dysuria, bladder spasm, and epithelial atrophy [4]. Pelvic RT’s adverse effects can manifest acutely or chronically, and studies have implicated inflammation and fibrosis as the primary mechanisms underlying these side effects. The rapid turnover of vaginal and vulvar epithelia renders them particularly vulnerable to RT-induced damage. Ionizing radiation generates reactive oxygen species (ROS), such as superoxide and hydrogen peroxide, through the reduction of oxygen. Acute radiation effects include vaginal congestion, mucus desquamation, and mucositis, while long-term consequences include vaginal thinning, narrowing, adhesions, and, ultimately, vaginal stenosis [3,5,7,9].

Although male rat models have been utilized to study the effects of pelvic RT on erectile physiology, research specific to female incontinence and sexual dysfunction is lacking [10,11]. To the best of our knowledge, this study represents the first female model of pelvic RT aimed at investigating both the acute and chronic injuries in vaginal and bladder tissues. The primary objective of this study is to assess the temporal effects of pelvic RT on vaginal, urethral, and bladder smooth muscle function and evaluate oxidative stress markers in vaginal tissues. At four weeks (4wk) and nine weeks (9wk) post-pelvic RT, we hypothesize that bladder, urethral, and vaginal smooth muscle contractility will increase. Furthermore, markers of oxidative inflammation are expected to be elevated acutely after pelvic RT. Enhancing our understanding of the temporal pathophysiology of RT-induced injuries will help to identify novel treatment strategies for improved management of pelvic RT-induced complications.

This work was previously presented at the American Urological Association Annual Meeting in Chicago, IL (May 2019) as a podium presentation, at the American College of Obstetricians and Gynecologists Annual Meeting in Nashville, TN (May 2019) as a poster presentation, and at the International Society for the Study of Women's Sexual Health Annual Meeting in Atlanta, GA (March 2019) as a podium presentation.

## Materials and methods

Animals 

A total of 24 female Sprague-Dawley rats, 12 weeks old (Charles River Laboratories, Wilmington, MA, USA), were housed in pairs with water and chow provided ad libitum. They were subjected to a 12:12 hour light/dark cycle. Experiments were conducted at 4wk or 9wk post-RT. Rats were randomly divided into three groups (n = 8/group): (I) Sham, (II) 4wk RT, and (III) 9wk RT. The Sham group was assessed at both 4wk and 9wk post-sham RT (n = 4/timepoint). Animal studies were approved by the Institutional Animal Care and Use Committee and followed the NIH guide for the care and use of laboratory animals.

Pelvic irradiation 

**Figure 1:**
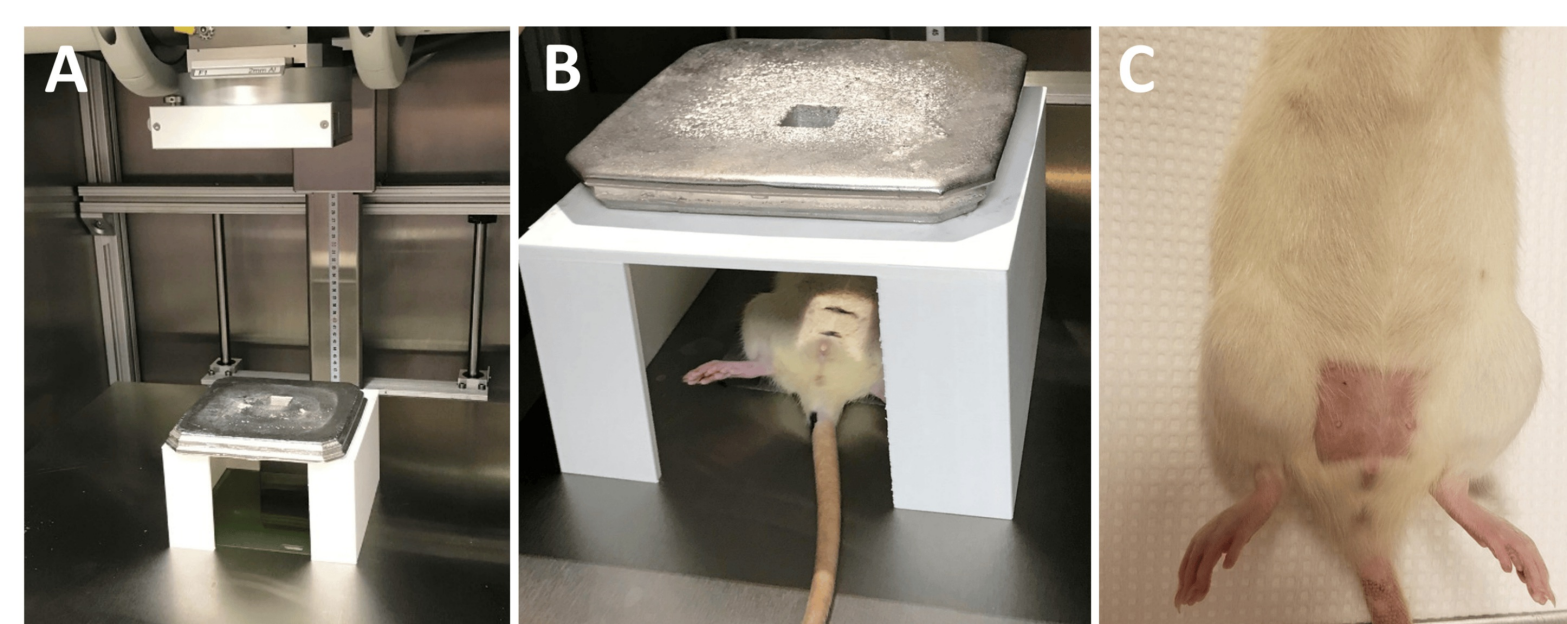
Pelvic irradiation experimental set-up (A) A 2 x 2 cm and a 3D printed table stand were used to create a limited radiation field. (B) The anesthetized rat was placed under the table stand and the Cerro blend collimator was aligned 0.5 cm superior to the external urethral opening. (C) At 9 weeks post-RT, visible hair loss was present in all animals.

Computerized tomography imaging was used to determine the location and depth of internal pelvic structures such as the bladder, cervix, and bowel. A 2 x 2 cm Cerro blend collimator was aligned 0.5 cm superior to the external urethral opening, and a 3D printed table stand was used to create a limited radiation field (Figure 1). Animals were anesthetized with a ketamine/xylazine mixture to receive pelvic radiation. Sham rats were also anesthetized and placed in the irradiator but did not receive the dose. The XRAD 320 X-Ray Biological Irradiator (Precision X-ray Irradiation, North Bradford, CT, USA) delivered a single dose of 20 Gy (320 kV, 12.5 mA, 50 SSD, 2.0 mm Al filter) to the rat pelvis within the designated radiation field. The dose was determined based on what tumor-adjacent normal tissue would receive. The dose was confirmed with equivalent dose calculators based on the Linear Quadratic Model (eviQ calculators, Australian NSW). The XRAD irradiator’s dose output was confirmed by a medical physicist using Gafchromic™ EBT-XD radiotherapy films (Ashland, Wilmington, DE, USA), Monte Carlo radiation transport code (EGSnrc) and MATLAB imaging software (MathWorks, Natick, MA, USA) [12].

Vaginal blood flow measurements

At 4wk or 9wk post-RT, vaginal blood flow was measured in anesthetized rats as previously described [13]. Vaginal cytology was performed to determine the rats’ estrous phase. The right carotid artery was cannulated for continuous measurement of mean arterial pressure (MAP) via a pressure transducer. The Type I implantable surface probe with an epoxy glass window (Transonic, Ithaca, NY, USA) was inserted approximately 1 cm into the vagina and angled to face the anterolateral vaginal wall. Increases in vaginal blood flow were measured by directly stimulating the lower pelvic ganglion at increasing voltages (2, 4, and 6 V) at 16 Hz, with a 5 ms duration, for 30 seconds, with a three-minute rest between stimulations. Vaginal blood flow was measured via a laser Doppler flowmeter (Model BLF22; Transonic) in tissue perfusion units (TPU, mL x min⁻¹ x 100 g⁻¹) and normalized to MAP. The area under the curve was measured to calculate total tissue perfusion over the 30-second stimulation.

Smooth muscle vasoreactivity

Following vaginal blood flow measurements, rats were euthanized via thoracotomy, and the vaginas, urethras, and bladders were carefully collected. Distal segments of the vagina were cut into 2 x 10 mm strips and mounted in a muscle strip tissue bath (820M, Danish Myo Technology A/S, Aarhus, Denmark). Vaginal strips were stretched to a resting tension of 5 mN for 60 minutes. The bladder was denuded of the mucosal layer, cut into 2 x 10 mm strips, and stretched to a resting tension of 4 mN for 30 minutes. Internal urethral sphincters (IUS) and external urethral sphincters (EUS) were also cut into 2 mm rings and stretched to a resting tension of 3 mN over 60 minutes. All tissues were maintained in a physiologic salt solution (in mM: 130 NaCl, 4.7 KCl, 14.9 NaHCO₃, 5.5 dextrose, 1.18 KH₂PO₄, 1.17 MgSO₄, 1.6 CaCl₂), aerated with 95% O₂ and 5% CO₂ (37°C, pH 7.4). Tissue reactivity and viability were assessed in response to a high potassium chloride solution (KCl, 120 mM) after the equilibration period.

Vaginal cholinergic and adrenergic-mediated contractions were measured via concentration-response curves (10^-9^ to 10^-4^ M) of carbachol and norepinephrine, respectively. Neurogenic contractions were measured via electric field stimulation (EFS) (Grass Instruments, Quincy, MA, USA) at increasing frequencies for 10 seconds, with two-minute rest intervals (20 V, 2 ms pulse width, 1-64 Hz). Vaginal strips were pre-contracted with norepinephrine, and relaxation was assessed with a nitric oxide donor, DEA-NONOate (10^-9^ to 10^-4^ M). Relaxation curves were normalized to the norepinephrine-induced precontraction force. Bladder cholinergic contractions were measured in response to carbachol (10^-9^ to 10^-4^ M). EFS-mediated contractions were recorded at increasing frequencies for 10 seconds, with two-minute rest intervals (EFS: 30 V, 0.3 ms pulse width, 1-64 Hz). Bladder contractions in response to carbachol (10^-9^ to 10^-4^ M) were repeated in the presence of atropine (10^-6^ M). IUS and EUS contractions to carbachol (10^-9^ to 10^-4^ M) were measured. IUS and EUS ryanodine-mediated contractions were measured in response to 40 mM caffeine. EFS-mediated contractions were measured at increasing frequencies for 10 seconds, with two-minute rest intervals (30 V, 0.3 ms pulse width, 1-48 Hz). IUS and EUS tissues were fixed in 4% paraformaldehyde, sectioned, and imaged to confirm that EUS contained circular striated muscles. All tissue bath measurements were recorded using LabChart 8 software and PowerLab acquisition hardware (AD Instruments, Colorado Springs, CO, USA). Wet weights of tissue strips were collected following all experiments. All compounds and drugs were obtained from Sigma Chemical Company (St. Louis, MO, USA).

Histologic analysis

The same animals were used for histological analysis. Bladder, vaginal, and urethral segments were fixed overnight in 4% paraformaldehyde, embedded in paraffin, sectioned, and imaged following Masson’s trichrome staining (Sigma Aldrich, St. Louis, MO, USA). Using freely available ImageJ software (NIH) and the freehand area selection tools, we measured vaginal epithelial thickness, bladder smooth muscle to total bladder wall surface area (the urothelial layer was digitally removed with the wand tool), and urethral smooth and skeletal muscle area [14]. The smooth muscle or skeletal muscle area is shown as a ratio to the total bladder or urethral surface area for each image. Three images per animal were analyzed and averaged.

Vaginal quantitative polymerase chain reaction (qPCR) 

Markers for inflammation or oxidative stress assessed included: interleukin-6 (IL-6), tumor necrosis factor α (TNFα), and NADPH oxidase 2 and 4 (NOX2, NOX4). Total RNA was extracted from vaginal samples (n = 8/group), purified (RNAqueous-Micro Kit; Invitrogen, Carlsbad, CA, USA), quantified (NanoDrop ND-2000c spectrophotometer; Thermo Fisher Scientific, Waltham, MA, USA), and reverse transcribed (SuperScript IV VILO Kit; Invitrogen, Carlsbad, CA, USA). Real-time qPCR was completed in triplicate using TaqMan Gene Expression Master Mix and TaqMan Gene Expression Assays (Applied Biosystems, Carlsbad, CA, USA) for each cDNA sample on the QuantStudio 6 Flex real-time PCR system (Applied Biosystems, Foster City, CA, USA). Gene expression assays for hypoxanthine phosphoribosyltransferase 1 (HPRT1) and mitochondrial ribosomal protein S12 (MRPS12) were used for internal controls, and the relative quantification of gene expressions was calculated against MRPS12 and HPRT1 using a 2^-∆∆CT^ method. 

Statistical analysis

Statistical analysis was completed using Prism 6 (GraphPad Software, San Diego, CA, USA). Groups were compared using a two-way analysis of variance (ANOVA) and Tukey’s multiple comparison post-hoc test for vaginal blood flow and tissue bath studies. A paired t-test was used to compare qPCR data groups. A p-value of <0.05 was used to indicate statistical significance. Data are expressed as mean ± standard error of the mean (SEM).

## Results

Animals

All irradiated rats experienced expected hair loss in the 2 x 2 cm area in which they received radiation (Figure 1C). All rats maintained a healthy weight following RT (4wk Sham: 299 g ± 15.7; 4wk RT: 306 g ± 14.5; 9wk Sham: 310 g ± 13.2; 9wk RT: 322 g ± 23.3, p > 0.05). Sham animals from both 4wk and 9wk groups did not exhibit any statistical differences and were combined into a single control group.

Vaginal blood flow decreases 4wk post-RT

**Figure 2:**
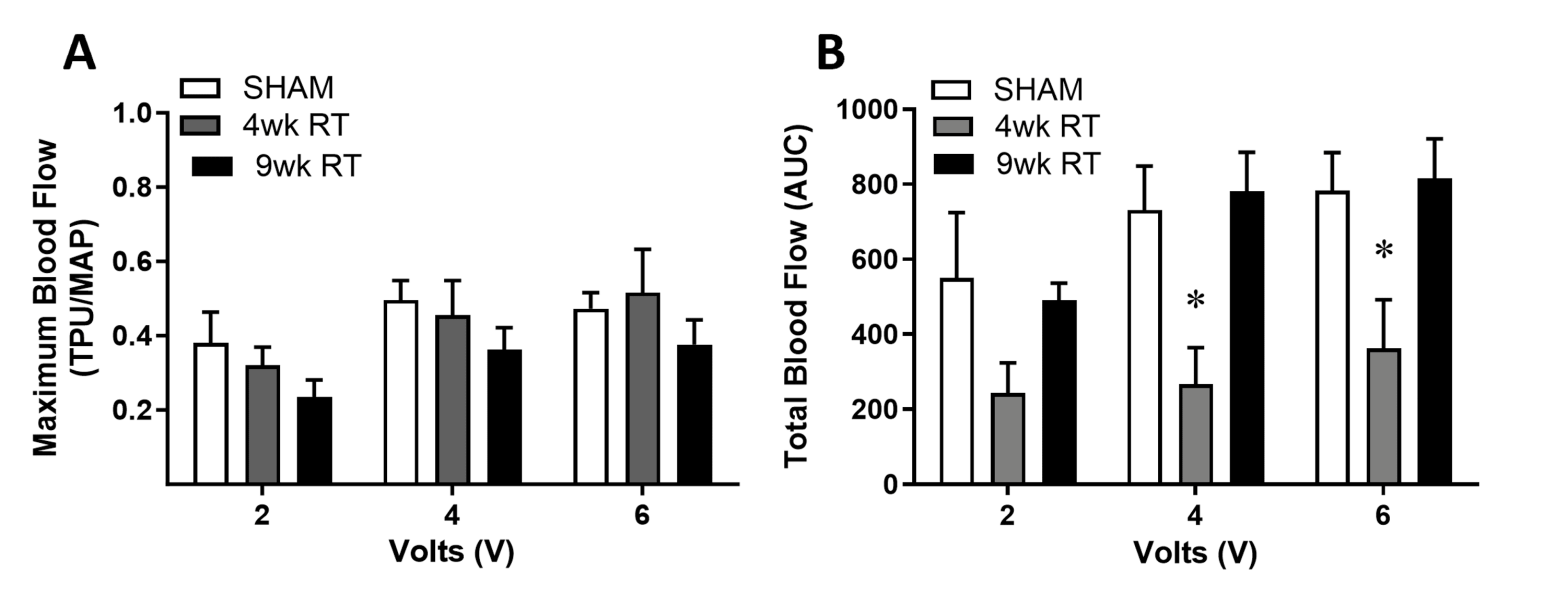
Pelvic nerve-stimulated total vaginal blood flow was decreased 4 weeks post-RT (A) Maximal blood flow was measured as a ratio of vaginal blood flow total perfusion units (TPU) to mean arterial pressure (MAP) and (B) the area under the curve (AUC) during stimulation was measured to assess total blood flow. Data are expressed as mean ± SEM. Two-way ANOVA indicated *p < 0.05 (*statistical significance) vs. Sham and 9wk RT; N = 7-8 per group. 9wk RT: Nine weeks post-radiation therapy; SEM: Standard error of the mean; ANOVA: Analysis of variance

**Figure 3:**
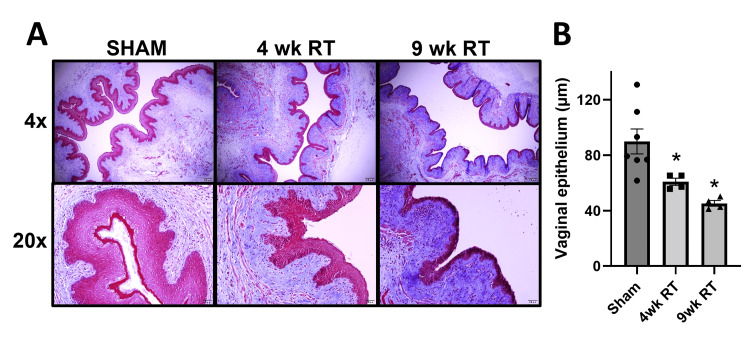
Pelvic radiation decreases vaginal epithelial thickness (A) Representative images of vaginal cross-sections stained with Masson’s trichrome show decreased epithelial thickness following radiation. Images were viewed with 4x and 20x objectives. (B) Vaginal epithelial thickness was measured using ImageJ. Data are mean ± SEM. One-way ANOVA indicated *p < 0.05 (*statistical significance) vs Sham; N = 4-6/group. 9wk RT: Nine weeks post-radiation therapy; 4wk RT: Four weeks post-radiation therapy; SEM: Standard error of the mean; ANOVA: Analysis of variance

There were no differences in maximum vaginal blood flow, normalized to MAP, in response to direct pelvic ganglion stimulation (Figure 2A). We also measured total blood flow, which represents the area under the curve, normalized to MAP. The 4wk RT group demonstrated a significant decrease in total vaginal blood flow, which returned to Sham levels by 9wk post-RT (Figure 2B). Additionally, vaginal epithelial thickness was decreased approximately 35% 4wk post-RT and more than 50% by 9wk post-RT (Figure 3).

RT increases vaginal smooth muscle tonicity

**Figure 4:**
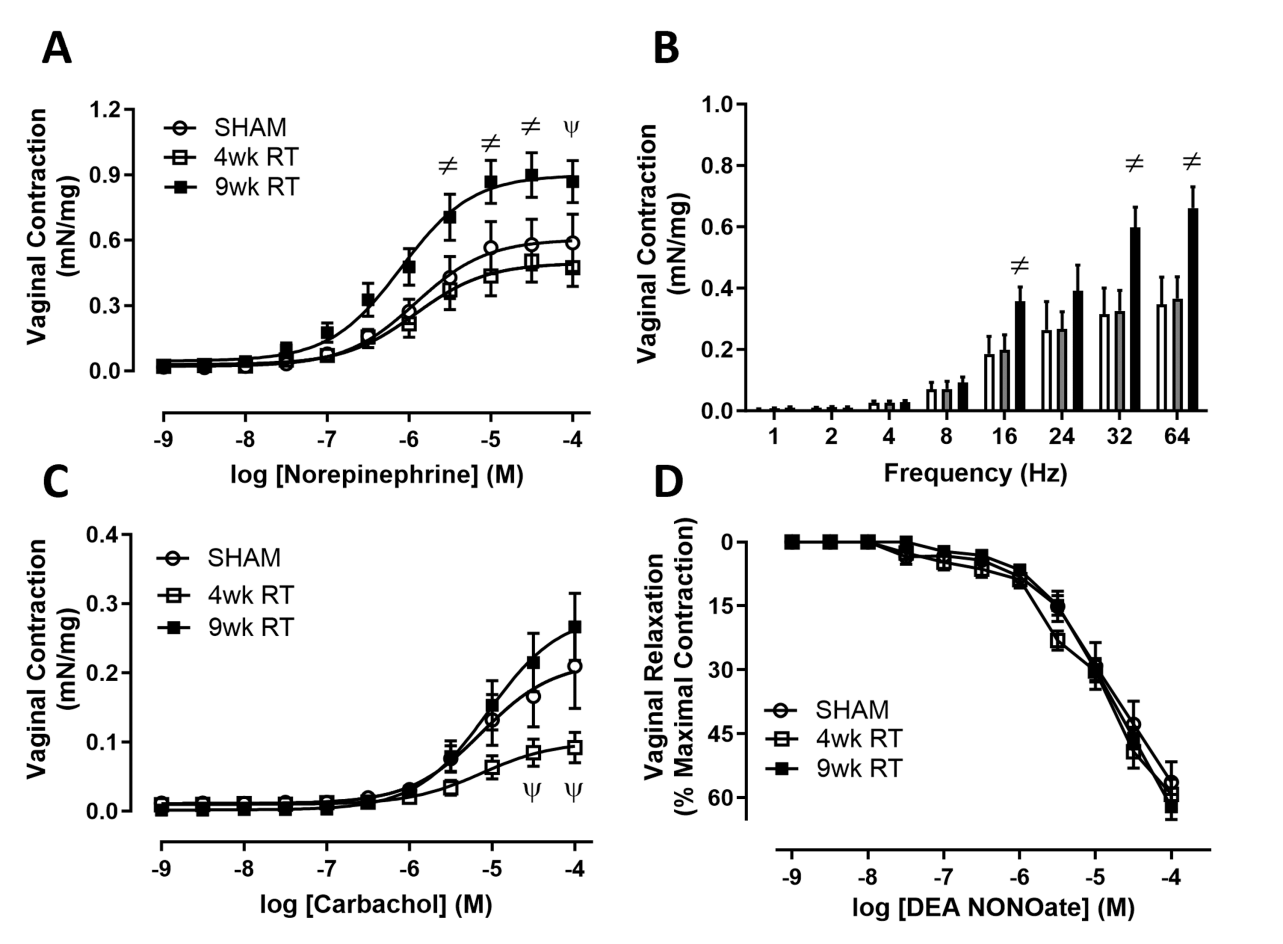
Pelvic radiation increased adrenergic and neurotransmitter-mediated vaginal contraction (A) Pelvic RT increased adrenergic-mediated and (B) electrical field-mediated vaginal contraction at 9wk post-RT. (C) Vaginal cholinergic-mediated contractions were lower at 4wk post-RT and recovered to Sham levels at 9wk post-RT. (D) Vaginal relaxations to a nitric oxide donor, DEA NONOate, were unchanged with RT. Data are mean ± SEM. Two-way ANOVA indicated *p < 0.05 (*statistical significance) vs. Sham and 9wk RT; N = 7-8/group. 9wk RT: Nine weeks post-radiation therapy; 4wk RT: Four weeks post-radiation therapy; SEM: Standard error of the mean; ANOVA: Analysis of variance; DEA NONOate: Diethylamine NONOate

Vaginal smooth muscle vasoreactivity was assessed ex vivo in a muscle strip tissue bath. At 9wk post-RT, adrenergic-mediated vaginal contraction increased (Figure 4A). Similarly, EFS-mediated neurogenic vaginal contractions were significantly increased at 9wk post-RT compared to the 4wk RT and Sham groups (Figure 4B). The 4wk RT group had decreased cholinergic contractions that returned to control levels by 9wk RT (Figure 4C). There was no difference in relaxation to a nitric oxide donor between any of the groups (Figure 4D).

Detrusor contractions impaired and persistent post-RT

**Figure 5:**
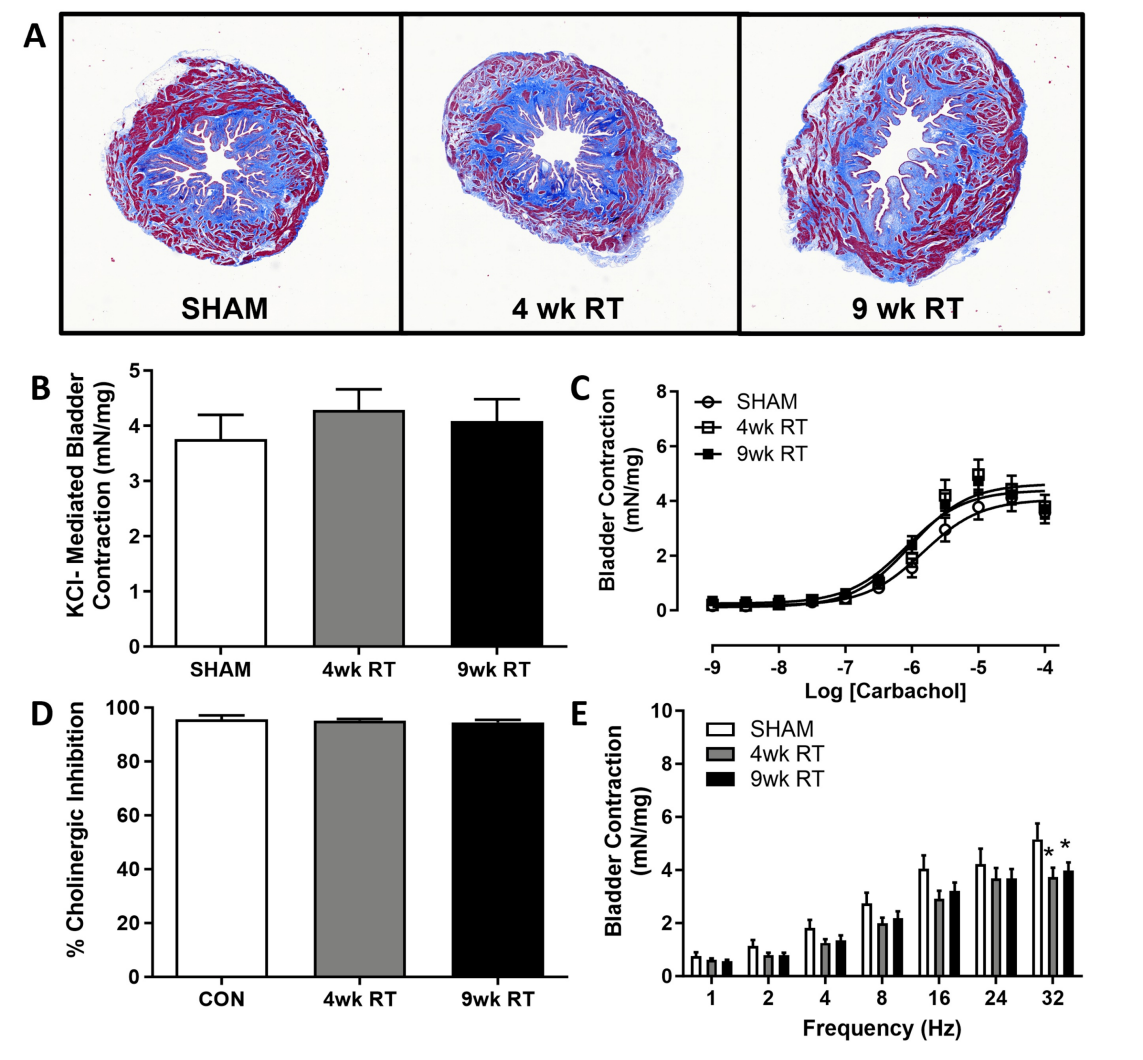
Nerve-mediated bladder contraction was lower following pelvic radiation (A) Representative images of bladder cross-sections stained with Masson’s trichrome (2x). (B) Maximal bladder contraction to high potassium chloride (KCl) was unchanged with RT. (C) Concentration-response curve to carbachol was also unchanged in bladders post-RT and (D) inhibition of carbachol at 3 x 10^-5 ^M to atropine was unchanged. (E) Bladder contraction to electrical field stimulation was decreased with RT at the highest frequency. Data are mean ± SEM. Two-way ANOVA indicated *p < 0.05 (*statistical significance) vs. Sham; N = 7-8/group. 9wk RT: Nine weeks post-radiation therapy; 4wk RT: Four weeks post-radiation therapy; CON: Contraction; SEM: Standard error of the mean; ANOVA: Analysis of variance

The bladder smooth muscle area was measured with Masson’s trichrome staining and was unchanged (Sham: 22.2% ± 0.99; 4wk RT: 20.1% ± 1.85; 9wk RT: 21.8% ± 1.37, data not shown; Figure 5A). Bladder contractions were unchanged in response to a high KCl solution or increasing concentrations of carbachol between the Sham and RT groups (Figures 5B-5C). Cholinergic inhibition with atropine caused similar inhibition of carbachol contraction across all groups (Figure 5D). Interestingly, there was a decrease in EFS-mediated neurogenic detrusor contractions at 4wk post-RT that remained decreased at 9wk post-RT (Figure 5E).

IUS and EUS contractions increase post-RT

**Figure 6:**
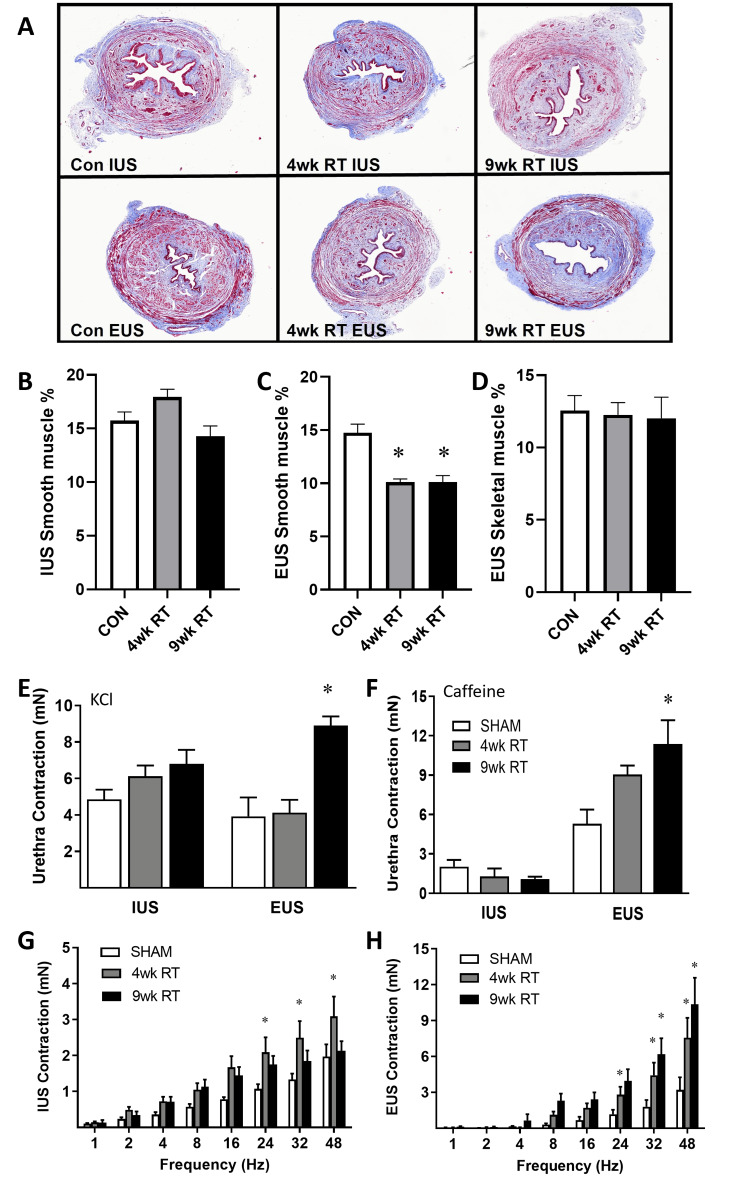
External urethral sphincter had increased contraction following pelvic radiation (A) Representative images of internal urethral sphincter (IUS) and external urethral sphincter (EUS) cross sections stained with Masson’s trichrome (2x). The percentage of IUS smooth muscle (B) was unchanged, but the EUS smooth muscle (C) was decreased with RT. EUS skeletal muscle (D) was no different post-RT. (E) IUS contraction to high potassium chloride (KCl) was similar; however, EUS KCl contraction increased at 9wk post-RT. (F) Caffeine-medicated contractions were unchanged in IUS rings but increased at 9wk post-RT in EUS rings. (G) Electrical field stimulated (EFS) contractions were increased at 4wk post-RT in IUS and (H) increased at both 4wk and 9wk post-RT in EUS. Data are mean ± SEM. Two-way ANOVA indicated *p < 0.05 (*statistical significance) vs. Sham; N = 7-8/group. 9wk RT: Nine weeks post-radiation therapy; 4wk RT: Four weeks post-radiation therapy; CON: Contraction; SEM: Standard error of the mean; ANOVA: Analysis of variance

Histological analysis confirmed that the IUS and EUS were correctly collected due to the presence of the skeletal muscle layer in the EUS rings (Figure 6A). The IUS smooth muscle area was unaffected by pelvic RT (Figure 6B), whereas the EUS had markedly decreased smooth muscle content at both 4wk and 9wk post-RT (Figure 6C). In contrast, the EUS skeletal muscle area was unchanged with pelvic RT (Figure 6D). In response to a high KCl solution, IUS contraction was unchanged following pelvic RT (Figure 6E). In contrast, EUS contractions to high KCl and caffeine were elevated at 9wk post-pelvic RT (Figure 6E). IUS contractions to caffeine were much lower than EUS contractions, confirming the appropriate segmentation of the EUS and IUS rings of the urethra (Figure 6F). Additionally, caffeine-induced contractions were unchanged in IUS segments but increased at 9wk post-RT in EUS segments. IUS nerve-mediated contractions were acutely increased in the 4wk RT group and returned to baseline in the 9wk RT group (Figure 6G). In contrast, neurogenic EUS contractions were increased in the 4wk RT group and remained increased in the 9wk RT group (Figure 6H).

RT causes acute increase in vaginal inflammation markers

**Figure 7:**
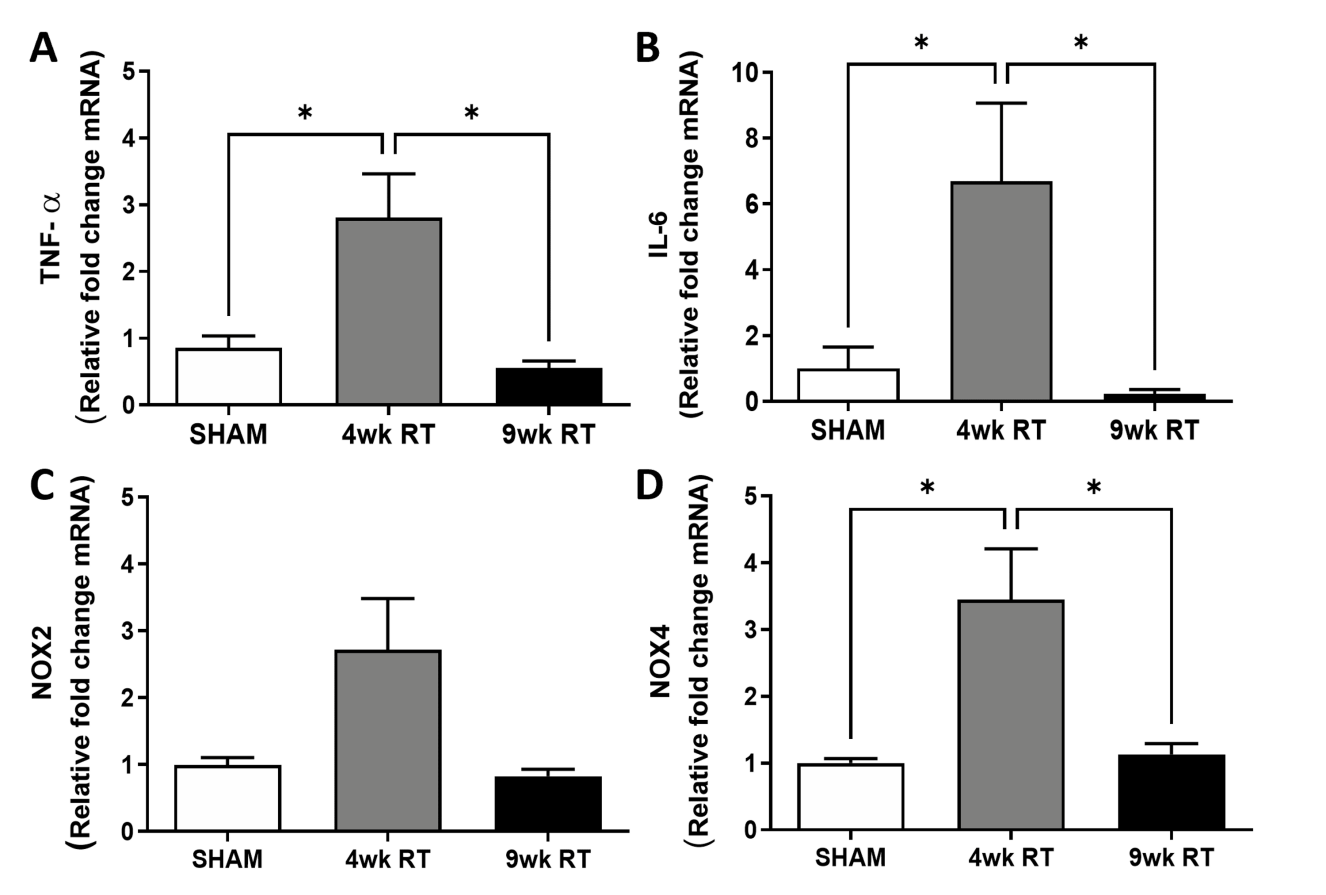
Temporal increase in vaginal gene expression of inflammatory markers after pelvic radiation (A) Representative images of internal urethral sphincter (IUS) and external urethral sphincter (EUS) cross sections stained with Masson’s trichrome (2x). The percentage of IUS smooth muscle (B) was unchanged, but the EUS smooth muscle (C) was decreased with RT. EUS skeletal muscle (D) was no different post-RT. (E) IUS contraction to high potassium chloride (KCl) was similar; however, EUS KCl contraction increased at 9wk post-RT. (F) Caffeine-medicated contractions were unchanged in IUS rings but increased at 9wk post-RT in EUS rings. (G) Electrical field stimulated (EFS) contractions were increased at 4wk post-RT in IUS and (H) increased at both 4wk and 9wk post-RT in EUS. Data are mean ± SEM. Two-way ANOVA indicated *p < 0.05 (*statistical significance) vs. Sham; N = 7-8/group. 9wk RT: Nine weeks post-radiation therapy; 4wk RT: Four weeks post-radiation therapy; SEM: Standard error of the mean; ANOVA: Analysis of variance; TNFα: Tumor necrosis factor α; NOX 2 and 4: NADPH oxidase 2 and 4; mRNA: Messenger RNA

Gene expression of vaginal inflammatory markers is acutely increased at 4wk post-RT and returns to baseline at 9wk post-RT (Figure 7). In the 4wk post-RT vaginal tissues, quantitative PCR analyses showed an increase in gene expression of NOX2, NOX4, IL-6, and TNFα. By 9wk post-RT, the gene expression of all inflammatory markers had returned to baseline.

## Discussion

Genitourinary dysfunction following pelvic RT has been extensively documented in clinical literature. Pelvic RT cancer patients frequently suffer from thinning of the vaginal epithelium, vaginal atrophy, stenosis, and bladder dysfunction [3-6]. However, the assessment of adverse effects has mainly relied on pelvic physical exams and subjective patient reports of symptoms [3,15]. In this study, we demonstrate changes in vaginal blood flow, urogenital smooth and skeletal musculature, and vaginal inflammation in both acute and chronic phases using a female rat model of pelvic RT. Our findings reveal an acute increase in ROS and inflammatory markers, along with a decrease in vaginal blood flow and vaginal epithelial thickness. In the chronic phase, we observed increased vaginal smooth muscle contraction, decreased detrusor contraction, and increased urethral sphincter contraction. To our knowledge, this is the first model to illustrate female-specific changes in both sexual and bladder physiology following pelvic RT. This preclinical model mimics the RT-induced adverse effects seen in the human condition and can be used to investigate prevention or recovery treatments for radiation-associated sequelae.

Inflammatory response and oxidative stress 

Radiation injuries occur in a time-dependent manner, characterized by an acute phase of inflammation and a chronic phase dominated by fibrosis [16]. Ionizing radiation leads to an acute increase in ROS, such as superoxide and hydrogen peroxide. These ROS activate inflammatory and cytokine pathways, which, in turn, increase ROS production and oxidative stress. In our study, we observed an acute increase in the vaginal gene expression of NOX2, NOX4, IL-6, and TNFα, which are involved in ROS generation and inflammation. Both NOX2 and NOX4 are ROS mediators regulated by the proinflammatory cytokine IL-6, and they directly contribute to oxidative stress and cell damage in radiation-exposed areas. Similar acute-phase markers have been identified in the intestinal mucosa following RT, including acute microvascular endothelial cell death, mucosal inflammation, and impaired epithelial renewal [17]. Our work suggests a similar decline in vaginal epithelial renewal, and therapeutics that benefit intestinal mucosal RT injury should be considered for pelvic organs.

Decreased vaginal blood flow 

Insufficient blood flow to inflamed vaginal tissue contributes to fibrosis, decreased vaginal mucosal thickness, and correlates with clinical experiences of dyspareunia or dryness in women following pelvic RT. Prior to this study, the development of vascular or smooth muscle dysfunction in vaginal tissue resulting from RT had not been thoroughly evaluated. Acute microvascular endothelial cell death following RT has been documented in various tissue types, including cardiac, intestinal, and penile tissues [11,17,18]. RT-induced ROS and inflammatory mediators perpetuate acute vascular injury, promoting thrombi, endothelial apoptosis, and oxidative stress [17-19]. As observed in our study, ROS and inflammatory markers decreased in the chronic period, coinciding with the return of vaginal blood flow. We hypothesize that the acute decrease in vaginal blood flow is due to ROS and inflammatory-mediated microvascular injury. We noted a return of vaginal vascular perfusion at nine weeks, suggesting that a longer time point may be necessary to observe the chronic vaginal vascular effects, as observed in male rat models of radiation-induced erectile dysfunction [11]. Additionally, the severity of RT injury is dose-dependent, and higher doses may yield different results. Future studies will investigate the effects of RT at different doses and over a longer time to provide a comprehensive understanding of chronic vascular changes.

Vaginal smooth muscle injury 

Coordinated vaginal smooth muscle contraction and relaxation are integral to the female sexual response [20]. Our findings align with our hypothesis that functional vaginal smooth muscle following RT would lead to an initial decrease in contraction, followed by a later increase. Acutely, vaginal tissue demonstrated reduced cholinergic contraction, but adrenergic and neurogenic contractions increased chronically. These findings correlate with the fibrotic pathogenesis of vaginal stenosis, which contributes to sexual dysfunction in women after cancer treatment [21].

The timeline for the development of vaginal stenosis lacks consensus in the current literature, with reported onset ranging from weeks to years following treatment [3]. We observed evidence of increased vaginal smooth muscle tone and epithelial thinning as early as 4wk post-RT treatment, suggesting that the onset of vaginal atrophy occurs well before it becomes clinically evident. This finding is supported by the work of Katz et al. and Rakhra et al., who observed similar outcomes, providing evidence of vaginal stenosis as early as two months following RT [22,23]. An intervention immediately following pelvic RT, prior to the onset of symptoms, may prove valuable in preventing vaginal stenosis.

Bladder and urethral injury

Pelvic RT can lead to bladder injury and disruption of urethral function [24]. In contrast to other studies assessing RT-induced bladder dysfunction, we are not directly irradiating the bladder. This study demonstrated that neurotransmitter-mediated detrusor contractions decrease acutely following pelvic RT. Marks et al. describe the dose-dependent nature of radiation injury to the bladder in mice [25]. Mice that receive greater than 20 Gy of bladder RT develop poor bladder compliance and decreased filling pressures acutely following RT, with a transition to fibrotic disease years later [25]. The 9wk post-RT timepoint of our study may not be late enough to demonstrate increased fibrosis and decreased bladder smooth muscle. Radiated IUS and EUS segments demonstrated a persistent increase in contraction following pelvic RT. In a previous study of detrusor underactivity, urethral contractility was also increased as a guarding reflex to prevent urinary incontinence [26]. Increased urethral contractions, combined with poor detrusor contractility, may lead to incontinence, urinary stasis, and chronic cystitis.

Limitations and future focus

The incidence of RT injury depends on the mode and dose of RT. In this study, we evaluated injury following a single 20 Gy dose of pelvic RT. Clinically, cancer RT typically involves multiple treatments over time, and future studies could benefit from evaluating injuries at various timepoints during a fractionated treatment regimen. Others have compared single versus fractionated radiation in radiation-induced brain injury in mice. Overall, the total dose was a more important parameter for brain necrosis, and no difference was evident in single versus fractionated delivery [27]. While this study is the first to evaluate the effects of RT on vascular blood flow to the vagina and urethral toxicity, the 9wk timepoint used may not have been long enough to evaluate long-term effects such as vaginal and urethral atrophy and fibrosis [15]. Neuromuscular and neurovascular injuries play a significant role in incontinence and sexual dysfunction [28,29]. Previous studies in male rat models of pelvic RT have demonstrated injury to the pudendal nerve and artery, contributing to RT-induced erectile dysfunction [30]. To further solidify the etiology and identify potential therapeutic targets, it is essential to evaluate nerve and vascular injuries in females following pelvic RT. Investigating cytokine production, immune cell response, and neovascularization of peripheral pelvic vasculature, as explored in RT-induced erectile dysfunction studies or in radiation-induced vascular diseases, holds promise for preventing chronic neurovascular injuries in females [30]. Additionally, in future studies, we will use protein analysis of inflammatory markers of interest to validate our gene expression data. Future research can focus on identifying the immune and inflammatory-mediated responses to nerve injury to discover new therapeutic targets specific to female genitourinary tissues.

## Conclusions

In female rats, pelvic RT acutely induces inflammation, decreases vaginal blood flow, and increases vaginal contraction. Bladder contractility decreases following RT, but EUS and IUS contractility both increase post-RT. These changes parallel the pathophysiological changes seen in vaginal and bladder tissue in irradiated clinical populations. Additionally, these data validate previous work showing RT causes vascular injury and support the role of ROS and inflammation in acute injury. Evaluation of chronic RT injury beyond nine weeks requires further investigation. The long-term survival of pelvic cancer patients is increasing, and the focus is shifting to prioritize quality of life following treatment. Identifying effective prevention and treatment of genitourinary injuries following RT is increasingly important. This model can be used to develop treatments for cancer survivors living with acute or chronic side effects of pelvic RT.
